# Gleanings from Our Exchanges

**Published:** 1872-11-01

**Authors:** 


### Gleanings from Our Exchanges.

### CLINICAL SIGNIFICANCE OF SPUTA.

Dr. A. J. Steele, of St. Louis, writes to the *Medical and Surgical Journal*, of that city:

We have not been able to diagnose pulmonary tuberculosis by means of the tubercle cell found in sputa; and while willing to believe that there is a tubercle cell, *sui generis*, which if found in expectorated matter would afford sufficient evidence of the presence of tubercle in the lung, still, practically, this is not known to be possible; for there are so many other elements going to make up the mass of the sputa, namely, epithelial, pus and mucous cells, granular matter, broken down and degenerated tissue, that our cell tubercle, if actually present, is so obscured and concealed as not to be recognized; again, if present, it has so undergone disintegration as to have lost its characteristics, and thus cannot be made available.

The microscope detects in expectorated matter but a single substance that may be considered pathognomonic, namely, elastic tissue. Its presence signifies lung destruction, brought about by inflammation and ulceration, the latter process not confining itself superficially to the mucous surface, but having extended deeper into the parenchyma itself.

The basement framework of those organs so beautifully adapted to the aeration of the blood, reminding one, with their open spaces and interstices, of sponge, is composed of elastic tissue, which, being of comparatively low vitality, *i. e.*, less vascular than most other tissues, is but slowly susceptible to the destructive process; and thus from ulcerative action fragments of it will be detatched and thrown off, still retaining, however, their histological arrangement, being thereby readily recognized under the glass. All sputa does not contain it, but when found it points unerringly to but one source, and thus its clinical importance.

While the appearance of these elastic fibrillæ is peculiar, yet the novice might confound them with the fibre of linen, silk, or cotton. Still, each have their characteristics, and a little practical observation will enable one to distinguish each from the other. So, also, the curved streaks of mucus so often present have been mistaken for elastic fibres. A drop of acetic acid at once decides the difference. Elastic fibres are curved, divide dichotomously, are of equal width, sharply contoured, and remain unaffected by acetic acid; once seen, are ever after recognized; best studied from section of healthy lung.

If from the sputa is taken a small quantity of the paler portion of the lumps, and if the grayish masses especially are examined on the slide, the elastic tissue will ordinarily be found. Specimens from different portions of the sputa should be subjected to the glass; a delicate hook to catch up, and scissors curbed on the flat to nip off the matter examined, are convenient. A drop of acetic acid, which dissolves for the most part the substances present, brings out the elastic fibres more decidedly. When much in doubt, it is well to boil the suspected expectoration in *liquor sodæ*. On cooling, the elastic tissue falls to the bottom of the settling glass, and can readily be removed with the pipette. A power of 300 is quite sufficient to exhibit the fibres.

Such a simple matter is this examination, that I am in the habit of subjecting the expectorated matter of most patients afflicted with lung trouble to it.

The special practical importance of this sign is its early recognition of the destructive changes taking place, before other signs indicate it; before even it is suspected, the microscope tells the certain story of disintegration of air vesicle walls. If diagnosis is much in the practice of medicine, surely an early knowledge of the facts is all the more important. "Had I seen the case earlier, or had I only known in time," so often remarks the medical man, "the issue would have been different." While undue prominence should not be given to the microscope, still it should hold its legitimate place in the means employed to diagnose disease. Too much neglected in the past, the day comes when it will be found a working tool in the hands of every physician.

Lung destruction associated with tuberculosis is much less amenable to treatment than when it is simply an inflammatory affection. The concomitant symptoms in the former case, the hereditary and personal peculiarities, with the history, enables it to be readily distinguished, and makes the prognosis guarded; while in the latter case, a good result would be expected, even though a cavity in the lung should have been found. Just here, and in conclusion, we cannot avoid the suggestion that old cicatrices found in post mortem examination of the lungs, and proclaimed to be evidence of pulmonary consumption cured or arrested,* have been proof merely of nature's repair of excavations produced by a simply ulcerative process entirely unconnected with the tubercular diathesis. These become the cases of so-called "spurious consumption."

### VIRCHOW ON COHNHEIM.

The position which Virchow would take on Cohnheim's discoveries has been a question of profound interest to the scientific world ever since the announcement of the "migration theory." For nearly two years the great master has withheld his opinion of the value and significance of the results claimed by Conheim, one of his most illustrious pupils, a fact which has given rise to much speculation on the part of those who "only swear by Virchow" as to the real import of Cohnheim's investigations. The publication of the new edition of "Virchow's Cellular Pathology raises the veil at last and squarely exposes his views of those more recent discoveries which seemed to threaten the foundation of Virchow's life-time labors.

Virchow still maintains, to the letter, his old opinions concerning the process of inflammation. It is observed in its simplest forms in non-vascular tissues where it induces nutritive and formative changes in the cells. In vascular tissues the vessels are brought into the same scale of changes ; white blood corpuscles escape from the vessels and the process is thereby complicated.

Concerning suppuration, Virchow expresses himself (p. 536) as follows:

"The more profound, properly ulcerative suppuration takes place regularly in connective tissue or its equivalents. There is first an increase in the size of the cells (connective tissue), the nuclei divide and proliferate in high degree for a considerable time. Very soon there follows then division of the cell elements themselves. In the vicinity of the region of irritation, in the place of the former individual cells, there are found double and manifold cells from which is developed usually a new formation, homologous in kind (hyperplastic connective tissue). Inwardly, where the cells were already filled with nuclei, masses of small cells appear which at first maintain the position and form of the former connective tissue corpuscles. Somewhat later are encountered roundish masses (depositions, abscesses) or diffuse infiltrations within which connective tissue is extremely spare, and as the deposition increases the connective tissue becomes more and more rare and soft. How great a share the immigration of white blood corpuscles takes in this procedure must be left to further and more accurate observation. Many modern and rather one-sided investigations, evidently deduced from false premises, have given a wrong interpretation to these processes. Yet this is all the more pardonable in that we ourselves have been just as one-sided hitherto in our consideration solely of cell-proliferation. As to the later course of the inflammatory process, it matters little whether the new cells are ascribed to proliferation or immigration."

And again, as a characteristic selection:

"The investigations into the immigration of the colorless blood cells are far from shaking my doctrine of cell derivation from previous cell, '*Omnis cellulæ cellulis;*' they have only served to confirm it. Much of error in interpretation has been corrected by this discovery, but the cellular principle receives from it an essential support. Whether a great part of the exudated cells arises directly from the blood, or whether these cells, as Stricker claims, divide and still further increase in the exudation, it matters not; the young breed, in either case, springs from previous cells.

"Plastic exudations are no longer plastic in the old sense of the word, and it is not the free plasma or fibrin which furnishes new cells by organic crystallization, not the intercellular substance which, as cytoblastema (Schwan's doctrine still of cartilage) produces new elements from itself, but it is the cell substance itself, the protoplasma of our day, from which, in the course of continuing proliferation, the organic entities are newly formed. The formative force (nisus formativus), the plastic power (vis plastica) belongs

*Watson's Practice, ed. 1858, p. 630.

still to pre-existent elements, not to the blastema, the succus nutritius. It is strange that there are a few who maintain that my whole theory of tissue construction was erected upon connective tissue; that it was only from connective tissue that new elements could be developed. At no time have I cherished such a view. I have at all times recognized the formative properties of the epithelial formations. It was I who first described the proliferation of the nuclei in the primitive muscular bundles, and of the capillaries during the process of irritation; more than all this, at a time when the colorless blood corpuscles were almost disregarded, I referred to these corpuscles the organization of the thrombus.* I am very far, then, from opposing myself, in envy, against any positive advance in our science; on the contrary, I hail every new discovery in this department as a new weapon in defense of my original views. —*The Clinic.*

### A CASE OF EXTRA-UTERINE GESTATION.

BY JEAN PAUL BONSIEUR.

*Rupture of the Large Intestine; Cesarian Section; Cure; by L. Adams, Surgeon to the Hospital of Sorocaba,* (Brazil).

Blandina de Barros, a mulatto, æt. 30, entered the hospital, October 13; she stated that her pregnancy was between the seventh and ninth months, and complained of pains across the epigastric region and in the loins. At the end of the ninth month these pains ceased, a general œdema invaded the body. We had, before her entrance, treated her for an ovaritis or metritis and so weakened her by medication that she was extremely reduced. The odor from the body was so great that no one was able to take care of her; she had an ulceration and a fistula near the umbilicus which permitted the escape of pus and excrement; and for a month pus was abundantly discharged from the rectum.

On her entry to the hospital M. Adams found her pregnant, but the uterus was empty; an injection thrown into the fistulæ returned by the same opening forcing out pus and fecal matter; on introducing a small pair of forceps he withdrew a bone, the radius of the infant, and afterwards a cuboid bone. Seeing the state of the patient, M. Adams decided on the performance of Cæsarian section. The patient was anæsthetised; he made a long incision of six inches through the abdominal walls and the adherent peritoneum. He tried to pass his finger through the opening, but met considerable resistance; he then cleansed the wound, finding a great quantity of false membranes adhering to the peritoneum, and a fibro-vascular tissue forming a second diaphragm enveloping the intestines and concealing the fœtus.

After a long dissection of the ovaries we arrived at the fœtus, which was extracted in a state of putrefaction, but with the loss of hands and feet which had escaped by the rectum, for the patient had, for near a month, loss of putrid matters by the rectum. Having cleansed the wound with a sponge impregnated with chloride of sodium, oil of camphor and benzoine, M. Adams found the uterus normal, and the only communication with the intestine was through the Fallopian tubes. The ovaries were nearly atrophied. There were three fistulæ from the rectum communicating with the abdominal cavity. The large intestine was divided and ulcerated; it was impossible to discover the termination, which was lost in the mass of tumor. The intestine is completely concealed in false membranes. The sphincter ani has lost all contractile power; to the left of the ovary we found a fistulous opening, which we recognized as the intestine. Dressed the wound and administered for the night an opium pill and an ounce of aromatic confection. In the morning the patient said it had been long since she had slept so well.

Oct. 14. The pulse 55; great debility. The patient had had a chill and complained of exhaustion. Ordered port wine and quinine. A great quantity of pus and fecal matter came through the incision.

From this time an improvement continued; on Nov. 10th cicatrisation was complete, and the feces found exit from the normal outlet. —*Gaz. Med. de Paris*, Aug. 10, '72.—*The Clinic.*

Transplantation of Black Skin. — M. Reverdin proposed the two following questions in his communication concerning skin grafts. 1, Is the graft an excitant to the action of the skin? 2, Is it the cells of the graft that multiply? M. Bryant adopts the latter opinion. A white man had a large ulcer of the leg. The surgeon transplanted upon it skin from a negro. The ulcer dimished in extent, and the black skin increased.

Apropos of this fact, the same author observes that it is better to transplant from the same individual than from another, as in this way danger of communicating cancer or syphilis is avoided.—*Gaz. Med. de Paris*, Aug. 24, '72.—*The Clinic.*

Recent Researches of Prof. Brown-Sequard on Epileptoid Convulsions.—At the stated meeting of the New York Pathological Society, Sept. 25th, Dr. Brown-Sequard gave, in a brief manner, some new facts in regard to the production of epileptoid convulsions in animals, especially the Guinea pig.

The first animal exhibited had been operated upon so as to expose the spinal cord. This operation in the Guinea pig, as was long ago demonstrated by the speaker, is almost invariably followed by convulsive attacks similar in three essentials to those of epilepsy in the human subject. By irritation of what the doctor calls the epileptic zone, the skin of the neck near the ear, convulsions can be produced at will; the movements last from thirty to sixty seconds. Animals so operated upon never recover. The injury is irreparable, and after some months death takes place. A recently discovered and very interesting fact with regard to the production of these convulsive movements in the Guinea pig, is that injury to other nerves will be followed by phenomena similar in all respects to those produced by injury to the spinal cord.

*Division of the Sciatic nerve* in the Guinea pig produces results similar to, if not identical with, those produced by injury of the spinal cord.

In this pig, remarked the professor, the sciatic nerve has been divided quite recently. I now irritate, by pinching the skin of the neck, and you notice that the animal is thrown into violent convulsions, of short duration.

Another animal was exhibited, in which the operation had been done some time ago. In this animal the convulsions could be produced with difficulty, as recovery was beginning to take place.

Another one was shown, nearly well, in which, after powerful pinching of the epileptic zone, no convulsions resulted. In these animals, recovery takes place when the divided nerve is re-united. You will observe in two of these pigs that the toes of the leg supplied by the divided nerve are gone; the animal itself has eaten them off. The explanation of this fact is very simple: sensation of the part is abolished, and the pig eats it, just as it would anything else that came in its way. Here you notice one toe is left; it is supplied with *sensation* by a branch of the crural nerve, and has, therefore, escaped destruction. I have said that animals, after a certain length of time, recover. This is shown by increased sensation in the epileptic zone, and by the falling off of the hair at the same spot. And now comes a very remarkable fact. These peculiarities or traits induced by this operation are *transmissable.* The young of the toeless father or mother, when also born without toes, are liable to convulsive seizures precisely similar to those of the parents, and recovery is preceded by falling off of the hair at the epileptic zone.

The facts show the wide range of phenomena produced when the nerves are injured, and no one observer, no matter how diligent, is capable of interpreting them. In conclusion, Dr. Sequard urged upon the younger members of the profession the necessity of aiding him in these investigations. The operations are simple, the results manifold and complex, and need careful observation and truthful record.

Dr. Sequard will, at an early date, give a series of lectures upon the results of his recent researches.—*Medical and Surgical Reporter.*

Healing of Ulcers by Serum.—I have had several opportunities, during the year 1871, of trying the method I explained the previous year, of healing ulcers by covering them with serum. I propose to enlarge the observations during the coming year, and vary the methods already employed. Several striking results have, however, been got. In one case, for example, a sore the size of a penny was healed in forty-eight hours; in another, one of three ulcers, each abou the size of a florin, was experimented upon, and closed in three days, while the other two, in all respects similar, but treated by "water dressing," remained unchanged. In another case four hours and a half sufficed to produce a thin bluish covering of epithelium, like the "healing line" along the edge of contracting sores. Considerable care is requisite to ensure success, as the fluid must be carefully protected from contact till it "sets." When these experiments are complete, I will give an account of them.—*Glasgow Medical Journal.—Canada Lancet.—The Clinic.*

Death of Phila. Physicians. — The last number of the *Phil. Med. Times* (Oct. 5, '72) records the death of Prof. Edward Parrish of the Phil. College of Pharmacy, author of the well known Parish's Pharmacy, and of Dr. George Pepper, Accoucheur to the Philadelphia Hospital.

A Nut for Vital Statisticians. — During the recent strike of the scavengers of Edinburgh, the mortality of that city fell to 15 per 1000, far below the ordinary average.

Ingrowing Nail.—I desire to add a mite to the evidence repeatedly given in the *Journal* that the removal of the nail (to my knowledge not always successful) is unnecessary.

About twenty years ago, I applied a bit of compressed sponge to afford temporary relief, and was delighted to find that it effected a radical cure. I make the sponge as solid as leather, by wetting, and then winding string very tightly round it, and drying it thoroughly. Of this, I cut a small pyramidial piece, less than a grain of rice. This I insert beneath the nail, and secure it by strips of adhesive plaster, applied longitudinally, to avoid compression. The sponge soon becomes moist, and swollen, keeping the nail from the irritated flesh. Any granulations should previously be destroyed with strong nitric acid. I have adopted this plan upon many occasions, and have never found it to fail. — *Benjamin Blower* — *Brit. Med. Jour.*, Sept. 21, '72.

